# Bioactive peptides from broccoli stems strongly enhance regenerative keratinocytes by stimulating controlled proliferation

**DOI:** 10.1080/13880209.2021.2009522

**Published:** 2022-01-27

**Authors:** Juan Nicolas-Espinosa, Lucía Yepes-Molina, Micaela Carvajal

**Affiliations:** Aquaporins Group, Plant Nutrition Department, Centro de Edafología y Biología Aplicada del Segura (CEBAS-CSIC), Campus Universitario de Espinardo, Murcia, Spain

**Keywords:** *Brassica oleracea*, plant biopeptides, wound-healing, skin, plant by-products

## Abstract

**Context:**

As the interest on the research of plant derived bioactive peptides (BPs) for nutraceutical, cosmeceutical and medical applications is increasing, in this work, the application of peptide derived from broccoli to keratinocytes was studied.

**Objective:**

We focussed on the characterization of different peptides hydrolysates from broccoli stems [extracted from total protein (E) and from membrane protein (MF)], and their activity when applied to human keratinocytes.

**Materials and methods:**

Peptide mixtures from broccoli stems (E and MF) were characterized by proteomics. They were applied to HaCaT cells in order to study cytotoxicity in a concentration range between 20 and 0.15625 µg of protein/mL and wound healing was studied after 24 and 48 h of treatment application. Also, proteomic and gene expression of keratinocytes were analysed.

**Results:**

Depending on the source, proteins varied in peptide and amino acid composition. An increased proliferation of keratinocytes was shown after the application of the E peptides mixtures, reaching 190% with the lowest concentrations, but enhanced wound healing repair with E and MF appeared, reaching 59% of wound closure after 48 h. At the gene expression and protein levels of keratinocytes, the upregulation of anti-oncogene p53 and keratinization factors were observed.

**Discussion:**

These results suggest that peptide mixtures obtained from broccoli augmented cell proliferation and prevented the carcinogenic, uncontrolled growth of the cells, with different mechanisms depending on the protein source.

**Conclusions:**

The results encourage the opening of new lines of research involving the use of *Brassica* peptides for pharmaceutic or cosmetic use.

## Introduction

Currently, plants from the Brassicaceae family are amongst the most cultivated crops worldwide. From this family, broccoli (*Brassica oleracea* L. var. *italica*) has a high economic impact, as shown by the 26 million tons of broccoli and cauliflower that were harvested worldwide (http://faostat.fao.org/). Apart from its benefits as vegetable, broccoli is recognized as containing several health-promoting bioactive compounds such as, glucosinolates, polyphenols or vitamin C, which add a great nutritional value to its edible part (Picchi et al. 2012).

However, the harvesting of broccoli crops results in a high amount of non-used plant materials or by-products, such as leaves and stems (Dominguez-Perles et al. 2010). Therefore, the use of byproducts in different industries could make the crop more affordable and more sustainable. In this way, the stems have been reported to contain a high concentration of proteins (Shi et al. 2019), but their use has not been explored thus far. Also, membrane vesicles from raw broccoli material were isolated in previous studies, and these were shown to contain a high concentration of proteins (Martínez-Ballesta et al. 2018), although the hydrolysation of these proteins was not investigated.

Presently, the interest on research on plant-derived bioactive peptides (BPs) for nutraceutical, cosmeceutical and medical applications is increasing. Peptides are defined as short amino acid chains usually ranging from 2 to 20 units with a molecular weight under 3 kDa. The characterization of plant-derived BP released after enzymatic hydrolysis has shown a wide range of different bioactivities (Maestri et al. 2016), and many plants have been studied, i.e., BP from legume plants, chickpea, soybean, pea, lentil, mung bean and mustard. In particular, these have been shown to be effective against microorganisms, in lowering cholesterol, antiviral, antithrombotic, antioxidant, antihypertensive effects, amongst others (Ariza-Ortega et al. 2014; Lemes et al. 2016; Hua et al. 2018). As plant-derived BP have shown to have a wide variety of bioactivities, it is thought that they could also have the capacity to induce diverse signal transduction mechanisms that could trigger the expression of regulatory genes (Maestri et al. 2016).

Whereas animal-derived BP are normally applied as single peptide formulations, plant-derived BP are mainly used in cosmetics as raw mixtures of enzymatic hydrolysates derived from proteins extracts (Apone et al. 2019). Therefore, the source (type of organism, organ, etc.) and type of extraction (buffered aqueous solution, alcoholic, etc.) has been reported to modulate the bioactivity of the final peptide mixtures (Apone et al. 2019).

New peptide drugs and mixtures are being developed successfully *in vitro*, for their future cosmeceutical applications such as dermal regeneration, collagen and elastin synthesis, and improved wound healing *in vivo* (Lupo and Cole 2007; Apone et al. 2019). For this reason, current research is now focussed on the effect of these BP on the skin, specifically in keratinocyte cells, which are the major cell type found in the epidermis (Eckert et al. 1997). The advantage of using these peptide hydrolysates in the skin formulations is their ability to easily penetrate to deeper layers of the skin due to their low molecular weight, which could act as an efficient delivery system itself (Badenhorst et al. 2014). BP could play important roles in cosmetic anti-ageing and therapeutic applications such as wound healing and burn treatment, reducing excessive scarring, fibrosis and inflammation (Ndiaye et al. 2012; Dia et al. 2014; Rodríguez-Carrio et al. 2014).

In order to study the mechanisms triggered by the action of any bioactive compound preparations, it is important to measure specific markers, such as the expression of pro-inflammatory cytokines, i.e., tumour necrosis factor α (TNF-α), CCL2 and interleukins (IL-1a, IL-6), and the cells should maintain normal cellular morphology and epidermal differentiation. In this way, p53 is an important anti-oncogene, and its measurement is therefore important to study tumorigenic responses (Pastore et al. 2011). For studies with keratinocytes, the aquaporin AQP-3 has been described to stimulate their proliferation and is involved in skin tumorigenesis (Hara-Chikuma and Verkman 2008; Wu et al. 2014). In contrast, proteins such as Bax are also highly studied in keratinocytes, as they could trigger cell apoptosis (Arend et al. 2015).

This work used a bioanalytical approach with the biopeptides obtained from broccoli stems, directly and from isolated membranes, to study their combined effect on keratinocyte proliferation with the use of a wound healing assay. In addition, the gene expressions of TNFα, AQP3, p53, IL6, IL1a, CCL2, Jun, Bax, β-FGF and ICAM-1, and proteomic profiling will be determined to elucidate the underlying mechanism for growth control.

## Materials and methods

### Plant source

Stems of broccoli, *Brassica oleracea* L. var. *italica*, cv Parthenon (commercial seeds from Sakata Seed Iberica, S.L.U., Valencia, Spain) were collected, during winter season of 2020, from a commercial farm located in the Region of Murcia (Spain) the day before being processed, where they were grown under Mediterranean climatic conditions.

### Isolation of peptides from broccoli stems

Fresh broccoli stems (200 g) were homogenized with 1:2 (w/v) of water to obtain the juice. The stem juice was filtered with nylon mesh with a pore diameter of 100 µm and after with qualitative filter paper (Hua et al. 2018). The juice was heated to 72 °C to flocculate the proteins. After protein flocculation, the extract was placed into the refrigerator (4 °C) overnight. The flocculated proteins were then collected after centrifugation at 2500×*g* for 30 min, after which the precipitate was lyophilized to freeze-dry the protein extracts (E).

For the microsomal fraction (MF), extraction samples of fresh broccoli stems (300 g) were sliced in small pieces and vacuum-infiltrated with a 1:1:6 (w/v) ratio of an extraction buffer (0.5 M sucrose, 1 mM DTT, 50 mM HEPES, 1.30 mM ascorbic acid, pH 7.5), and 0.5 g of PVP (polyvinylpyrrolidone). After 10 min, samples were homogenized and filtered through a nylon mesh with a pore diameter of 100 µm. Then, the filtrate was centrifuged at 10,000×*g* for 30 min at 4 °C; the supernatants were collected and centrifuged for 35 min at 100,000×*g* at 4 °C. The pellet obtained was resuspended in 300 µL of 5 mM potassium phosphate buffer containing 0.25 M sucrose, pH 6.5. The protein concentration of stem extracts, the total protein from whole homogenate extract, and the MF proteins, were measured using the Bradford method (Bradford 1976).

Next, the protein extracts were hydrolysed with trypsin at 0.02% (w/v), and the protein suspension containing 0.35 mM Na_2_SO_4_ and 0.68 mM EDTA was incubated for 4 h at 50 °C and pH 8–8.5 with trypsin (0.01% w/v) (Hua et al. 2018). The reaction was stopped by incubating the mixture at 90 °C for 10 min followed by centrifugation at 6000×*g* for 5 min. The supernatants were filtered through a 0.22 µm membrane to sterilize the mixture.

### HaCaT cell culture and extracts application (peptides from broccoli stems)

HaCaT cells, the spontaneously immortalized human keratinocyte line (Boukamp et al. 1988), were cultured in Dulbecco’s modified Eagle’s medium (DMEM) (4.5 g/L glucose) supplemented with 10% foetal bovine serum (FBS), 1% penicillin–streptomycin and 1% l-glutamine at 37 °C, and 5% CO_2_. The HaCaT keratinocyte cell line was routinely cultured into 75 cm^2^ culture flasks. The subcultures were carried out every three days when the cells reached 70–90% confluence.

### Cell viability (MTT assay)

The effect of different concentrations of two class of peptides mixtures (E and MF) on the viability of HaCaT cells was determined with the MTT [3-(4,5-dimethylthiazol-2-yl)-2,5-diphenyltetrazolium bromide] assay (Mosmann 1983). Cells were plated at a concentration of 3200 cells/well in 198 µL of DMEM complete medium in a 96-well plate and cultivated at 37 °C and 5% CO_2_ until 60–70% of confluence. Afterwards, 2 µL of different 100X peptides mixtures were added to the wells (each sample was repeated six times), and incubated for 24 and 48 h. Cell viability was determined by adding 200 µL of MTT (1 mg/mL in DMEM) after completely removing the medium from the wells, and incubated for 4 h at 37 °C and 5% CO_2_. The MTT solution was removed and 100 µL of DMSO were added and the plate shaken. The absorbance was recorded at 570 nm on a microplate reader (BMG Lab Technologies, Fluostar Omega, Offenburg, Germany). The cell viability (%) of treated cells was calculated as follows:

(1) Cell viability (%)= (Abs570 nm)sample(Abs570 nm)control × 100

### Scratch wound healing assay

HaCaT cells were seeded into 24-well plates at 100,000 cells/well in complete medium (without phenol red) and incubated until 80% confluence was reached. Then, the medium was removed, and a vertical and a horizontal scratch was created in the centre of the well using a sterile tip. The cells were subsequently washed with PBS and treated with the peptide mixtures E and MF **(**both 0.3125 and 0.15625 µg protein/mL). The groove was monitored and photographed immediately, 24 h and 48 h after the scratch was made using a phase-contrast microscope (Leica DMRB, Eugene, OR) with ×5 magnification. The migration was evaluated in the residual area of the groove, and five different fields were counted for each condition. The wound area was calculated by tracing a line along the border of the wound using ImageJ software (Bethesda, MD) (Schindelin et al. 2012), and the percentage of wound closure was calculated using the following equation:

$$\text{Wound closure }(\%)=\frac{\text{wound area }(0\;h)-\text{wound area }(\text{X h})}{\text{wound area }(0\;h)}\;\times 100$$

### Quantitative real-time RT-PCR

HaCaT cells were seeded in six-well (95,000 cells/well) until the cells reached 60–70% confluence and treatments (E and MF) were applied for 24 h. Then, RNA was extracted from HaCaT cells using the NZY Total RNA Isolation kit (NZYtech, Lisboa, Portugal) as indicated by the manufacturer. RNA concentrations were determined with a Nanodrop 2000 Spectrophotometer (Thermo Fisher Scientific, Waltham, MA). cDNA was synthesized from 500 ng of RNA using the High-Capacity cDNA Reverse Transcription Kit (Applied Biosystems, Foster City, CA) according to manufacturer’s protocol. Real-time PCR analysis was performed in an Applied Biosystems QuantStudio 7500 Real-Time PCR system (Thermo Fisher Scientific, Waltham, MA) in 10 μL volumes, using the 2X Power SYBR Green PCR Master Mix (Applied Biosystems, Carlsbad, CA) with ROX as the passive reference dye. Volumes and concentrations for SYBR Green reaction mixes were 5 μL SYBR Green reaction mix, 500 nM forward and reverse gene-specific primers (Table 1) and 200 ng of DNA template. Amplification conditions were: 2 min at 50 °C, 10 min at 95 °C, followed by 40 cycles of 15 s at 95 °C, and 1 min at 60 °C. The amplification was performed on three independent samples for each treatment (biological replicates), and triplicate reactions were carried out for each sample (technical replicates) in 96-well plates. The transcript levels were calculated using the 2^–ΔΔCt^ method (Livak and Schmittgen 2001).

**Table 1.** Primer sets used for qRT-PCR.

Target name	Accession no. NCBI	Forward primer (5′ → 3′)	Reverse primer (5′ → 3′)
β-Actin	NM_001101	AAATCTGGCACCACACCTTCTAC	ATAGCACAGCCTGGATAGCAAC
IL-6	NM_000600	GTGTGAAAGCAGCAAAGAG	CTCCAAAAGACCAGTGATG
TNFα	NM_000594	TCCTTCAGACACCCTCAACC	AGGCCCCAGTTTGAATTCTT
IL1-α	NG_008850	TGGCTCATTTTCCCTCAAAAGTTG	AGAAATCGTGAAATCCGAAGTCAAG
p53	AH002919.2	CCTCAGCATCTTATCCGAGTGG	TGGATGGTGGTACAGTCAGAGC
BAX	NM_138764.5	TCAGGATGCGTCCACCAAGAAG	TGTGTCCACGGCGGCAATCATC
βFGF	NG_029067.2	TCAAAGGAGTGTGTGCTAAC	ATACTGCCCAGTTCGTTTC
CCL-2	NM_002982.4	AAGCAGAAGTGGGTTCAGGA	TAAAACAGGGTGTCTGGGGA
ICAM-1	NM_000201.3	CCCATGAAACCGAACACAC	ACTCTGTTCAGTGTGGCACC
AQP3	NG_007476.1	CTTGAGCATCCACTGACT	GGGTGAGGGTAGATAGGG
JUN	NG_047027.1	AAGTAAGAGTGCGGGAGGCA	GGGCATCGTCATAGAAGGTCG

### Protein analysis in HaCaT cells

#### Preparation of total protein extracts

For total protein extraction, HaCaT cells were seeded into six-well plates (95,000 cells/well) until the cells reached 60–70% confluence, and treatments E: 0.3125 and 0.15625 µg protein/mL, and MF: 0.3125 and 0.15625 µg protein/mL; were applied for 24 h. After that, the cells were trypsinized and collected by centrifugation for 5 min at 1000×*g*. Then, the pellet was washed with PBS buffer and centrifuged (5 min, 1000×*g*). Then, the pellet was resuspended in lysis buffer (150 mM NaCl, 1% Triton X-100, 50 mM Tris–HCl pH 8, supplied with protease inhibitor solution 1×, SIGMAFAST), and the cells were lysed on ice for 30 min with stirring. The lysate was harvested into a 1.5 mL centrifuge tube and centrifuged at 16,000×*g* for 20 min at 4 °C. Then, the supernatant was transferred into a new tube for protein quantification using the Bradford method (Bradford 1976) and LC–MS/MS analysis.

#### LC–MS/MS analysis

Before LC–MS/MS analysis, the samples were passed through a PD Spin Trap G-25 desalting column (GE Healthcare, Chicago, IL) to remove compounds which may interfere with the identification of proteins by LC–MS/MS. Then, the samples were mixed with 100 μL of 50 mM ammonium bicarbonate (pH 8.3) containing 0.01% ProteaseMax (Promega, Madison, WI). After that, the samples were reduced by adding 100 μL of 20 mM DTT at 56 °C for 20 min. Then, alkylation was performed by incubation with 100 μL of 100 mM IAA for 30 min, in the dark at room temperature. Digestion was performed by incubation with 1 μg of trypsin (1:100, w/w) for 3 h, at 37 °C. The samples were dried in a speed vacuum concentrator and resuspended in 20 μL of water/acetonitrile/formic acid (94.9:5:0.1). Then, they were injected into an Agilent Advance Bio Peptide Mapping HPLC column (2.7 μm × 100 mm × 2.1 mm, Agilent Technologies, Santa Clara, CA) thermostatted at 55 °C and with a dual electrospray (AJS-Dual ESI). The experimental parameters were set in MassHunter WorkstationData Acquisition software (Agilent Technologies, Santa Clara, CA), as described in Martínez-Ballesta et al. (2018). The MS data were processed with the Spectrum Mill MS Proteomics Workbench (Agilent Technologies, Santa Clara, CA) using human sequences from the Uniprot database (www.uniprot.org) (Bateman 2019). Protein function and location were determined with the Gene Ontology database (The Gene Ontology Consortium et al. 2019). The identification of plant functional peptides from both extracts (E, MF) was carried out with PlantPep DB libraries (Das et al. 2020).

Protein quantification data analysis was performed using protein intensities (Aguilan et al. 2020) with some modifications and the Reactome pathway browser (Jassal et al. 2020).

### Statistical analysis

The statistical analysis was performed using SPSS software (v.26; Chicago, IL). Statistical differences between the experimental groups were determined via a post hoc analyses (Duncan, Student's *t*-test, Dunnett). In all the tests, probability values of *p* < 0.05 represented statistically significant differences.

## Results

### Stem peptide characterization

**Figure 1.**
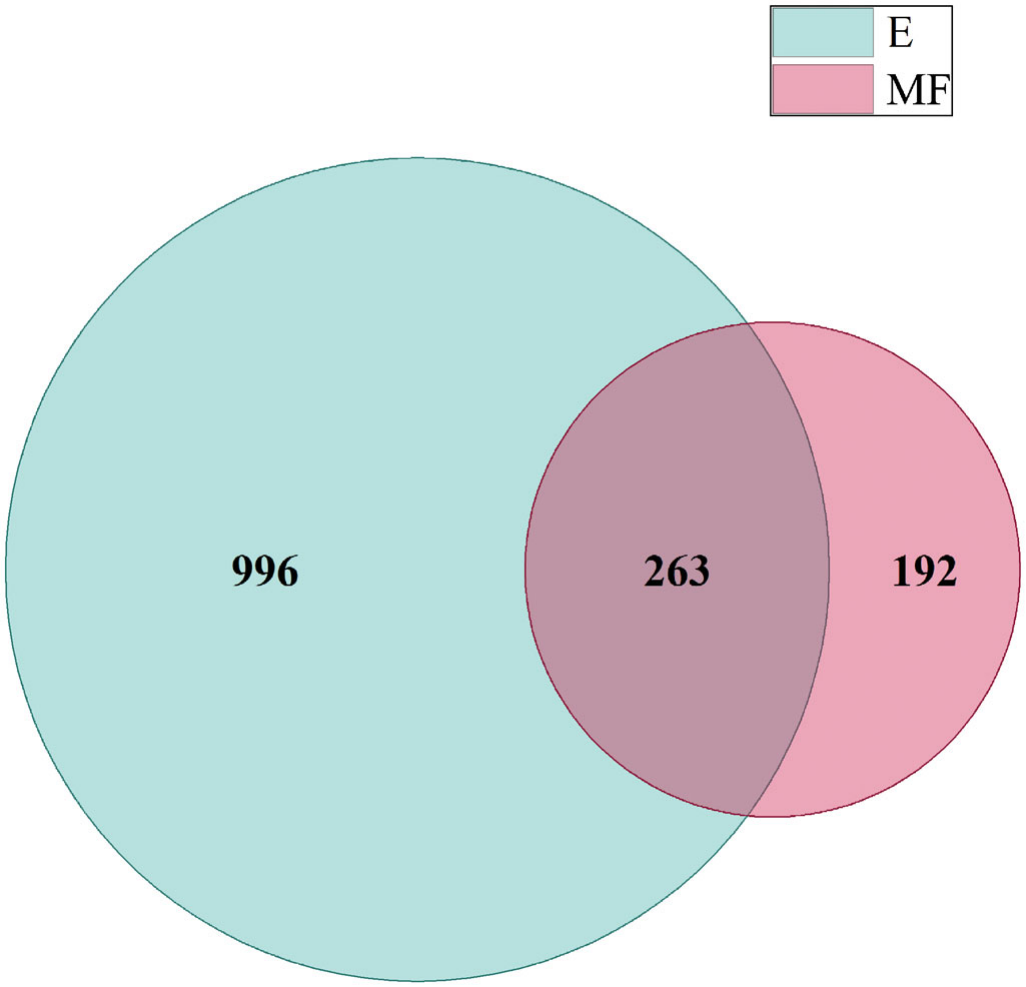
Venn diagram of peptide sequences identified in both protein hydrolysates, total protein extract (E) and microsomal fraction extraction (MF).

Broccoli stem extracts, total homogenate protein and MF extracts (E and MF, respectively) were trypsinized and analysed with LC–MS/MS to determine the set of peptide sequences that comprised each mixture. Peptide characterization of the different protein extractions showed that a total number of 1256 peptide sequences were identified in total protein extract (E), while in the MF extraction, the number of identified peptides were lower, 455 (Figure 1). In spite of this, 263 common peptide sequences were found in both extractions, E and MF. Additional analysis of the peptide extract was performed finding low amounts of organic acids, sugars and chlorine (Supplemental Table 1).

In addition, the relative amino acid composition (%) of the peptides was also analysed (Table 2), with some differences observed between the two extraction methods, such as the concentration of isoleucine (Ile), which was found to be higher in the MF peptide mixture. Also, the aromatic amino acid tyrosine (Tyr) was found in a larger concentration in E than in MF extracts, being present in the extracts as 1.71 and 1.28%, respectively. Lastly, a statistical difference in methionine (Met) concentration was also found. While the concentration was 1.59% in the E extracts, in MF extracts this was higher, 2.32%. Moreover, not only were differences between single amino acids found; differences between amino acid groups were also observed (Table 2). In particular, both hydroxylic and sulphur-containing amino acid groups, were observed in greater concentrations in the MF extraction mixture, MF.

**Table 2.** Relative amino acid composition of protein hydrolysate extracts, total protein extract (E) and microsomal fraction extraction (MF).

			E	MF
Amino acids	Aliphatic	Ala	8.65 ± 0.05	11.02 ± 2.24
		Gly	7.55 ± 0.23	7.64 ± 0.52
		Ile	7.33 ± 0.1**	8.43 ± 0.1**
		Leu	12.55 ± 0.02	8.65 ± 1.62
		Pro	5.5 ± 0.16	5.52 ± 0.86
		Val	10.03 ± 0.14	10.22 ± 0.26
	Aromatic	Phe	3.44 ± 0.04	2.97 ± 0.25
		Trp	0.53 ± 0.05	0.46 ± 0
		Tyr	1.71 ± 0.02**	1.28 ± 0.04**
	Acidic	Asp	6.45 ± 0.2	5.45 ± 0.34
		Glu	7.8 ± 0.26	5.28 ± 1.33
	Basic	Arg	3.26 ± 0.2	3.41 ± 0.44
		His	1.25 ± 0	1.12 ± 0.34
		Lys	5.07 ± 0.03	5.8 ± 0.46
	Hydroxylic*	Ser	5.58 ± 0.01	7.61 ± 1.29
		Thr	5.72 ± 0.09	7.29 ± 0.59
	Sulfur-containing*	Cys	0.02 ± 0.01	0 ± 0
		Met	1.59 ± 0.05*	2.32 ± 0.16*
	Amidic	Asn	3.07 ± 0.07	2.85 ± 0.11
		Gln	2.91 ± 0.14	2.67 ± 0.66

Student’s *t*-test statistical analysis (*p < 0.05 and **p < 0.005). Data are mean ± SE.

In order to identify possible functional peptides in the extracts, a complete screening of the sequences was performed, comparing them with the peptide sequences found in the PlantPep DB (Supplemental Table 2). As a result, eight perfectly matched functional peptides were identified in the extracts. In fact, all the peptides were present only in the total protein from the homogenate extract (E), in contrast with the MF extract, where only four functional peptides were found (Table 3). The functional peptides found were divided into two functional categories: antioxidant and antimicrobial activities, and were identified in Apiaceae as *Angelica sinensis* L. (ginseng), in Poaceae as *Oryza sativa* L. (rice) and *Triticum dicoccum* L. (emmer wheat). In particular, AGFAGDDAPR, AGLQFPVGR, IGGIGTVPVGR and QTVAVGVIK were found in both samples, E and MF, and were described to be have antioxidant activities, with these peptides isolated from *A. sinensis* (Wang et al. 2016); while ESTLHLVLR, NSSYFVEWIPNNVK and STTTGHLIYK were found only in the E samples, and were described as well as antioxidants but unlike the others, these were first described in *T. dicoccum* (Babini et al. 2017). Lastly, the ARFEELNMDLFR peptide, first described in *O. sativa,* was identified in the E mixture, but unlike the others, this peptide had a proven antimicrobial activity against *Porphyromonas gingivalis* (IC_50_=120 µM) and *Candida albicans* (Taniguchi and Ochiai 2017).

**Table 3.** Bioactive peptides described in the bibliography identified on the peptide mixtures (E and MF) and peptide function shown.

Peptide sequence	Presence		ID plant PepDB	Plant source	Peptide function	Peptide function description
	E	MF				
AGFAGDDAPR	x	x	PPepDB_3072	*Angelica sinensis*	Antioxidant	Used to delay ageing process in *C. elegans* through antioxidant activities independent of dietary restriction (Wang et al. 2016).
AGLQFPVGR	x	x	PPepDB_3073			
IGGIGTVPVGR	x	x	PPepDB_3308			
QTVAVGVIK	x	x	PPepDB_3565			
ARFEELNMDLFR	x		PPepDB_3568	*Oryza sativa*	Antimicrobial	Used in promotion of health and/or the treatment of diseases. It is also used as antimicrobial, endotoxin-neutralizing, arginine gingipain-inhibitory and/or angiogenic activities (Taniguchi and Ochiai 2017).
ESTLHLVLR	x		PPepDB_3155	*Triticum dicoccum*	Antioxidant	Shows significant antioxidant activity, i.e., able to scavenge superoxide anion and hydroxyl radicals, organic nitro-radicals (ABTS, DPPH) and to inhibit lipid peroxidation (Babini et al. 2017).
NSSYFVEWIPNNVK	x		PPepDB_3524			
STTTGHLIYK	x		PPepDB_3626			

### Viability test, MTT

**Figure 2.**
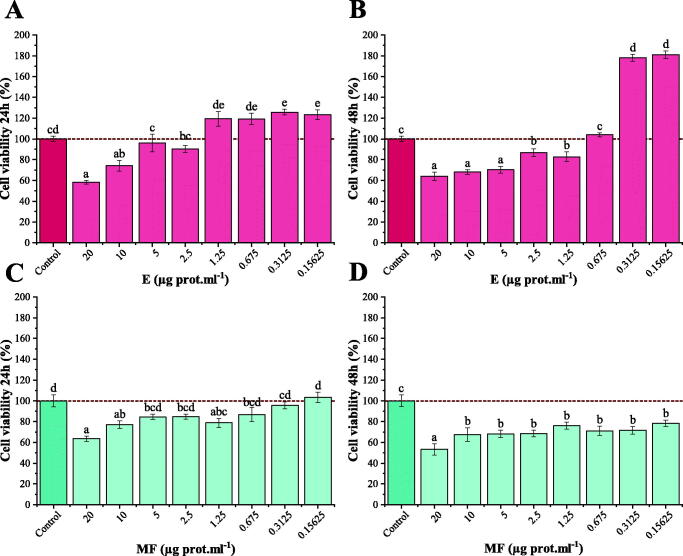
Cell viability test. HaCaT keratinocytes viability assay when treated with a concentration range (20–0.15625 µg protein/mL) of each peptide mixture (E and MF) after 24 h and 48 h. (A) Cell viability with different concentrations of E mixtures after 24 h. (B) Cell viability with E extracts after 48 h. (C) Cell viability with different concentrations of MF mixtures. (D) Cell viability with MF extracts after 48 h. Different letters indicate significant differences between groups after one-way ANOVA and Duncan’s test (*p* < 0.05).

Immortalized HaCaT keratinocytes, a non-malignant cell line, were treated with a wide range of concentrations of both peptide mixtures, total protein (E) and MF, to determine their cytotoxicity. Eight different concentrations of each E and MF extracts were tested; from 20 to 0.15625 μg of protein per millilitre (Figure 2). Cell viability was measured 24 (Figure 2(A,C)) and 48 h (Figure 2(B,C)) after treatment application. As expected, higher concentrations of E and MF (20–10 μg/mL) had a significant negative effect on cell proliferation, decreasing cell viability down to 60–80% in the first 24 h when compared with control, untreated cell culture conditions. However, no statistically differences were observed in cell viability after 24 h when utilizing 5, 2.5 or 1.25 μg/mL of the E mixture. In fact, a statistically significant cellular growth occurred after the application of lower concentrations of the E mixture in the first 24 h after the application of the treatment. Likewise, cytotoxicity at 48 h was also measured. In accordance to what occurred in the first 24 h, higher concentrations of the E mixture (20, 10 and 5 μg/mL) caused a significant decrease in cellular proliferation, around 60–70%, when compared with untreated cell culture. In contrast to the 24 h analysis, when 2.5 and 1.25 μg/mL of E were utilized, cell viability was also reduced (85–80%). No differences were observed when using E 0.675 μg/mL, whereas the two lowest E concentrations produced a boost in cellular growth, almost doubling the proliferation of untreated cells (190%).

In addition, the MF peptide mixtures showed no statistical differences when applied at concentrations ranging from 5 to 0.15625 μg/mL, as measured after the first 24 h. In fact, 1.25 μg/mL of MF had a higher cytotoxicity, reducing cell viability. Cell viability measured at 48 h revealed an overall decrease in cell viability with all the concentrations assayed (68–80%), with the lowest value found when 20 μg/mL of MF was applied (55%).

### Wound healing assay

**Figure 3.**
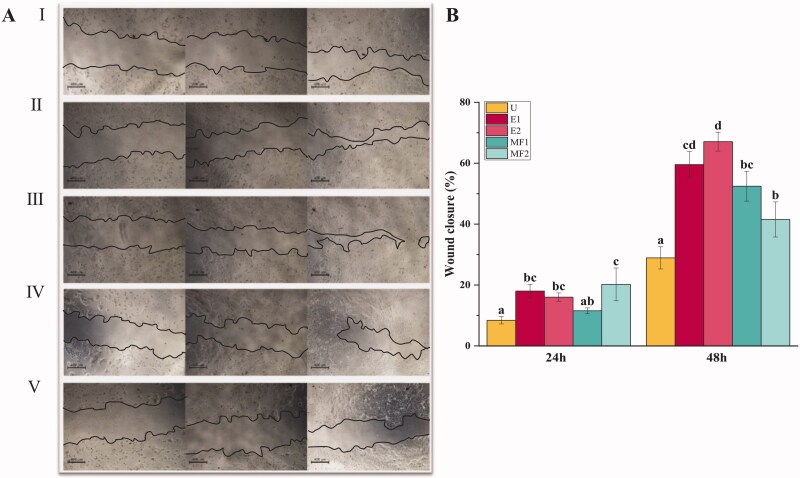
Wound closure analysis. (A) Phase contrast microscopy images of wound closure in keratinocytes cultures at different times (0, 24 and 48 h) when treated with peptide mixtures: control (I), E1 (II), E2 (III), MF1 (IV) and MF2 (V). Black highlighted lines define wound edges. (B) Relative wound area after 24 and 48 h of mixtures application. Different letters indicate significant differences between groups after one-way ANOVA and Duncan’s test (*p* < 0.05).

The results obtained from the cell proliferation assay led us to choose the two lowest concentrations of E and MF peptide mixtures (0.3125 and 0.15625 μg/mL) for the subsequent analysis. As shown above, proliferation assays showed some positive effects in cell growth when 0.3125 and 0.15625 μg/mL of the E mixture were utilized, with these differences being more evident after 48 h from the application of the mixtures. Consequently, HaCaT cells were grown to 80% confluence and scratched with a 20–200 μL pipette tip. Cell cultures were incubated in complete medium with 0.3125 and 0.15625 μg/mL of E (E1 and E2) and MF (MF1 and MF2) mixtures, respectively, and wounds were photographed 0, 24 and 48 h after treatment (Figure 3). Wound closure was measured, and statistical analyses were performed. Some significant differences were found between the treatments and control after 24 h of the application of the mixtures, with all the treatments (except for MF1) showing a statistically significant higher wound closure than untreated cells. While the untreated cells reached 8.4%, E1 and E2 extracts showed wound healing percentages of 16 and 18%, and MF2 of 20.2% after the 24 h treatments where applied. However, these changes in wound closure area where most evident after 48 h from the start of the experiments. Therefore, all the treatments showed higher statistically significant wound closure areas than untreated cells, with the E2 mixture being the most effective treatment, reaching a 67% wound closure area. Nevertheless, the E1, MF1 and MF2 treatments also showed positive results in wound closure area after 48 h treatments were applied (59.5, 52.5 and 41.5%, respectively), while the control cells reached a wound closure area of 29.2%.

### Quantitative PCR analysis, gene expression

**Figure 4.**
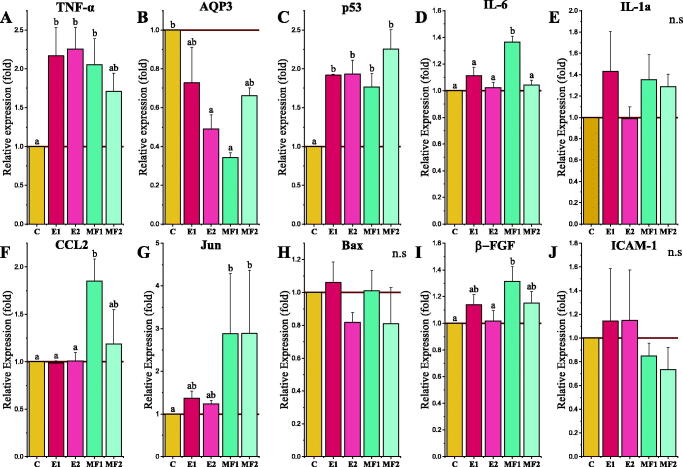
RT-qPCR analysis. Relative gene expression of TNFα (A), AQP3 (B), p53 (C), IL6 (D), IL1a (E), CCL2 (F), Jun (G), Bax (H), β-FGF (I) and ICAM-1 (J) in keratinocytes after the application of E1, E2, MF1 and MF2 peptide mixtures. Data are mean ± SE (*n* = 3). Different letters indicate significant differences between groups after one-way ANOVA and Duncan’s test (*p* < 0.05).

We compared the responses of HaCaT keratinocytes to the different treatments (E1, E2, MF1 and MF2) by analysing the gene expression of a set of genes that were especially involved in cell proliferation, apoptosis and pro-inflammatory activities. Out of all the genes that were analysed, TNF-α, AQP3 and p53 showed the most remarkable significant differences with control cells (Figure 4). TNF-α and p53 where overexpressed 24 h after the treatments were applied. Furthermore, the expression of TNF-α was two-fold in all the treatments, just as p53. On the contrary, the expression of AQP3 was down regulated when the treatments were applied, especially with the E2 and MF1 mixtures, reaching 0.48- and 0.34-fold expression. In addition, IL-6, CCL2, Jun and β-FGF seemed to be upregulated in keratinocytes treated with the MF1 and MF2 mixtures, being more prominent in the MF1 treatments. Alternatively, no statistical differences on gene expression were observed in IL-1a, Bax or ICAM-1.

### HaCaT proteomic profiling

**Figure 5.**
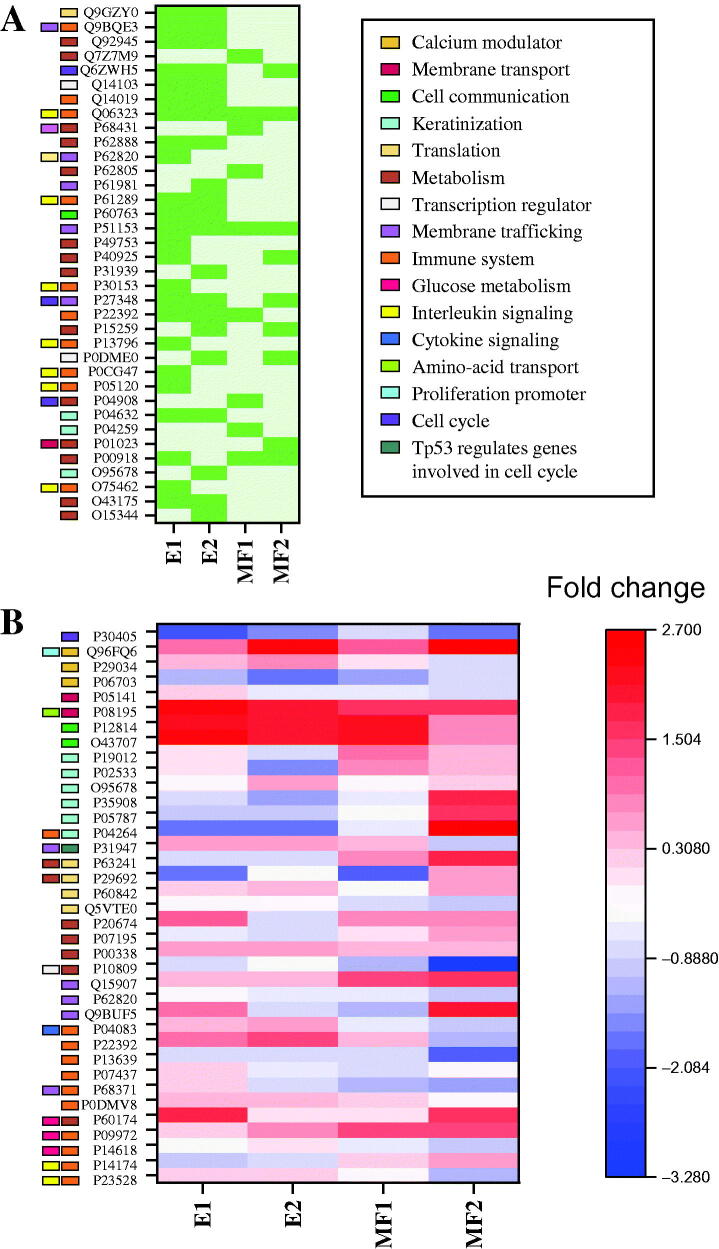
Keratinocytes proteomic profile differences. (A) Differential protein presence in keratinocytes when treated with different mixtures (E1, E2, MF1 and MF2) compared with control keratinocytes cultures. (B) Differential quantification of keratinocytes proteins between cells treated when compared with control, colours indicate quantity fold change.

After 48 h from the application, a cellular protein extraction was carried out to create a proteomic profile of the treated and untreated keratinocytes. First, a proteomics profiling was conducted, considering the molecular function and biological process of the protein groups, respectively ((Supplemental Tables 3 and 4). Comparative analyses of protein composition and abundance were conducted, with some differences observed between the control and the treatments, and between treatments as well (Figure 5(A)). When analysing the protein types as compared with the control, we found a high presence of proteins associated with the immune system and interleukin signalling, in particular involving interleukins 12 and 17 pathways, being more significant in the E1 and E2 treatments, where the most remarkable differences were observed. In contrast, both MF mixture treatments resulted in a lesser change in protein composition when compared with untreated HaCaT cells. In the same manner, some proteins associated with membrane trafficking, and involved in the cell cycle, were found in the protein extracts of treated cells, being more present in both E extracts. Nevertheless, when protein abundance was analysed, some expression patterns were found. The expression of some proteins was overall down regulated when the treatments were applied (P30405, P06703, P10809 and P13639), as compared with untreated keratinocytes. However, other protein batches seemed to be more abundant in all cases when the treatments were applied (Q96FQ6, P08195, P12814, O43707, P00338, Q15907, P60174 and P09972). A special case was found for P08195, a 4F2 cell-surface (CD98hc) antigen heavy chain, as it was found to be highly overexpressed in all the treated cells, in particular with both E mixtures.

Nevertheless, some patterns in protein abundance between treatments were found, as shown by the differential protein concentration between the E and MF mixtures (Figure 5(B)). Some proteins were overexpressed when the E mixtures were applied (P29034, P04083 and P22392), which mainly involved the immune system, while other proteins appeared to be less abundant than in untreated cell cultures (P35908, P05787 and P04264).

Alternatively, MF mixtures also showed some patterns in protein abundance, although these were less common as compared with those found when the E treatments were applied, such as P19012, P02533 (keratin, type I cytoskeletal 15 and 14, respectively) and P63241 (eukaryotic translation initiation factor 5A-1), which were more abundant in MF-treated cells than in untreated ones. On the contrary, no down-regulation of protein expression patterns was found only when the cells were treated with the MF mixtures. Additionally, P31947, a 14-3-3σ protein, was found to be more abundant in E1, E2 and MF1, in treated rather than untreated cells.

## Discussion

Presently, the research interest for new potential bioactive compounds derived from plants is increasing. Moreover, the utilization of the byproducts from plant crops, such as leaves and stems from broccoli, opens the possibility of transforming the industry. In the present work, the study and characterization of the extractions of proteins directly from stems or from isolated membranes from MF is derived from the promising results obtained with other plant proteins (Tokudome et al. 2012; Lemes et al. 2016; Maestri et al. 2016; Burnett et al. 2018; Sanguigno et al. 2018).

The different protein extraction methods resulted in different peptide mixtures and amino acid compositions. In fact, the MF peptide mixture had a higher relative concentration of Ile when compared with E. This could be explained by the source of the proteins extracted from the MF, as previous studies with membrane proteins obtained a higher presence of hydrophobic amino acids in the Brassicaceae family (Garcia-Ibañez et al. 2021). In the same manner, Met levels were found to be higher in the MF extracts when compared with the E mixtures. It is well known that peptides composed of sulphur-containing amino acids have a wide range of pharmacological activities, in particular when disulphide and thioether bonds are formed, and are shared in multiple physiological reactions, such as antioxidation and nitrosylation, amongst others (Góngora-Benítez et al. 2014; Zhao and Jiang 2018). On the other hand, the E extracts showed a higher concentration of Tyr, an aromatic amino acid with antioxidant activity (Gülçin 2007).

Additionally, previous studies determined that some peptide sequences similar to those identified in our peptide mixtures, showed antioxidant activities (Table 3). In this report, the peptides obtained from *A. sinensis,* improved survival of *Caenorhabditis elegans*, by decreasing endogenous ROS level and increasing antioxidant enzyme activities such as SOD and CAT (Wang et al. 2016; Babini et al. 2017). Moreover, a single peptide sequence (ARFEELNMDLFR, Table 3) is also shown to have antimicrobial activity (Taniguchi and Ochiai 2017). In this way, it has been reported that antimicrobial peptides also contribute to keratinocyte wound repair as additional effect (Guaní-Guerra et al. 2010). Although a few peptide sequences were identified as having biological activity when compared with previous studies, there seems to be a high number of BP sequences in the mixture, due to the overall activity found when applied to cell cultures, but no individual characterization has been made. In fact, it has been recently reported that peptide mixtures from stems and leaves of broccoli plants have an antioxidant capacity with a hypolipidaemic effect when they were applied in relatively high concentrations, thus having more antioxidant activity than soy bean plant peptide mixtures (Hua et al. 2018). However, the mechanism of expression that provided the observed bioactivity needs to be elucidated.

In our study, we focussed on the effect of these mixtures on cell cultures, specifically, human keratinocytes, for evaluating the bioactivity of these peptide mixtures. Only when E was applied at the lowest concentrations, an increase in the keratinocyte proliferation rate was observed (reaching 190% of the proliferation rate) at 48 h, but a slight reduction was observed after MF application. In spite of this, when these concentrations of MF and E were tested on the wound healing assay, both (with the two lowest concentrations) produced an increase in the wound closure process at 24 h, with this being more evident 48 h after the administration of the mixtures. As expected, the E mixtures performed the highest wound healing rate, with the E2 mixture showing the most positive impact in wounded keratinocyte cultures. Therefore, to understand the molecular mechanisms that took place when the mixtures were applied to the cell cultures gene expression and proteomics analyses were performed. Our results showed an enhanced expression of the TNF-α transcript in keratinocytes after the application of the E1, E2 and MF1 peptide mixtures (Figure 4(A)). TNF-α is a widely studied cytokine that is mainly involved in immune response, inflammation and apoptosis, but also tissue remodelling, cell motility and cell cycle processes (Locksley et al. 2001). It also plays a crucial role in the control of skin cells, where it is the main cytokine regulator in psoriasis, dermatitis, drug-associated eruptions and other inflammatory diseases, also taking part in angiogenesis during the wound healing process as described in skin after an injury (Kondo and Ohshima 1996; Banno et al. 2004; Wijaya et al. 2020). Even so, the RT-qPCR results showed an increase higher than twofold of the TNF-α transcript levels when keratinocytes were treated with E1, E2 or MF1 mixtures (Figure 4(A)). However, no statistical differences were observed in interleukins and pro-inflammatory markers transcripts levels when the treatments were applied (Figure 4(D,E,J)), except for IL6 and CCL2 with the MF1 treatment.

TNF-α is also associated with tissue repair and extracellular matrix (ECM) remodelling, cytoskeletal changes, cell migration, keratinocyte differentiation and cell-fate control, being key in keratinocytes’ wound healing (Sivamani et al. 2007). Furthermore, TNF-α promotes tissue repair by inducing actin cytoskeleton regulators, integrin and adhesion molecules (Banno et al. 2004). This type of protein was found in the proteomic profiling of treated keratinocytes (Figure 5(B)), where proteins related with cellular motility, migration and cell integrity were found to be more abundant when the keratinocytes were treated with all four different mixtures (P12814, α-actin-1; O43707, α-actin-4; P04083 and annexin A1), especially α-actin-1 and α-actin-4, being almost twofold more abundant than untreated keratinocytes.

TNF-α is well-known to be a suppressor of human keratinocyte growth (Wang et al. 2019). However, our results showed an increased rate of cell proliferation and wound healing when the keratinocytes were exposed to the different mixtures, although an increase in TNF-α mRNA transcripts was observed. The upregulation of TNF-α was measured in the first 24 h after the application of the treatments, and could be the initial response of the keratinocytes to the treatments, thus resulting in an inflammatory cell state. In this way, only a significant increase in IL6 and CCL-2 expression was observed when cells were treated with MF1. Therefore, after the application of the two lowest concentrations of the E and MF mixtures, an increase in cellular proliferation was observed when E1 and E2 were tested, while a decrease of 20% in the cellular viability was observed with the same concentrations of MF, 24 h after the start of the treatments. However, all the treatments resulted in incremented wound healing. This apparent controversy could be explained by the inflammation response triggered in MF treated cells that could lead to a migratory effect, as reported by Diegelmann and Evans (2004). However, these authors reported that beyond certain levels of inflammation, a collapse of cells led to cell necrosis. Therefore, the complexity of the inflammatory response previous to proliferation and migration in wound healing (Braiman-Wiksman et al. 2007) revealed that the activation of interleukins by MF was positively related to wound healing but not to normal growth.

In addition, the APQ3 transcripts were also affected by the application of the different peptide mixtures, as shown by an overall reduction in expression, with these changes being more evident in keratinocytes treated with E2 and MF1 mixtures, reaching reductions of 0.50 and 0.35 in their expression, respectively, when compared with control cells. AQP3 is a membrane protein that functions as a water and glycerol transporter in the basal layer of the epidermis, and is strongly expressed in keratinocytes (Sougrat et al. 2002). AQP3 dysregulation has been associated to some skin diseases such as psoriasis when its expression was reduced, while an upregulation has been reported to be involved in skin tumorigenesis (Verkman et al. 2008). This evidence suggests the important role of AQP3 in the correct operation of the keratinocytes, and be highly important in wound healing processes. Our AQP3 expression results could lead us to hypothesize that a reduced water permeability and decreased uncontrolled cell migration was related to the healing process (Martinotti et al. 2019). On the other hand, an increase in AQP-3 expression levels could alter the cellular phenotype, involved in cancer progression via signalling (Verkman et al. 2008; Marlar et al. 2017).

In contrast, p53 was observed to increase with all four treatments with its expression almost double when compared with control cells. p53 acts as a tumour suppressor protein, and protects the genomic integrity of the cells (Kruse and Gu 2009). The expression of p53 is involved in ageing, and an overexpression of p53 in mice was shown to induce premature ageing phenotypes and the arrest of cellular proliferation (Tyner et al. 2002), being a good candidate marker in our experiments (Choudhary et al. 2017). Despite the expected reduction in cellular growth, our results showed an increase in cellular proliferation and wound healing. These results showed a clear advantage, resulting in an enhanced error-free proliferation controlled by p53. Together, the expression patterns observed (TNF-α, AQP3 and p53) when the peptide mixtures were applied seemed similar to those when the cells were exposed to UV radiation and the machinery works to prevent UV radiation damage (Chaiprasongsuk et al. 2019). Therefore, the peptide mixtures could protect cells from stress.

Furthermore, the overexpression of the proto-oncogene Jun, observed in MF1 and MF2-treated keratinocytes, could have revealed an important role in epidermal wound healing processes. As it has been reported that keratinocytes lacking Jun were unable to migrate or elongate in cell cultures at the border of wound closure assays and that an overexpression accelerates wound healing (Li et al. 2003; Yue et al. 2020) was a gene marker selected by our experiments. In this way, our peptides could increase Jun expression mainly in the MF samples. In addition, the activity of Jun has been shown to decrease PPAR-γ gene expression, which acts as a transcription factor for AQP-3 (Bae et al. 2019). Therefore, the downregulation observed in AQP-3 in MF1 and MF2-treated cells could be the result, of the upregulation of Jun of these cell cultures, which could be related to a slight reduction in normal growth and the activation of wound healing as well.

In contrast, no statistical differences were observed in the Bax and ICAM-1 gene expressions in neither one of the treatments. Since Bax is a marker of cellular apoptosis, not finding differences with the control cells could lead us to think that the application of the mixtures had no effect over cellular apoptosis increasing cell dead (Arend et al. 2015).

The proteomic profiling of the treated cells also revealed a pro-cellular motility and migration pattern which promoted epithelial wound repair. Necrosis markers P30405 and PIPF, which play a crucial role in cell death, were also reduced (Figure 5(B)). Also, as expected, p53 negatively regulates the expression of protein S100-A6 (P06703), involved in the inflammatory response, regulation of calcium binding proteins, and cellular proliferation and apoptosis (Otterbein et al. 2002; Donato et al. 2017). Another member of the S100 proteins, S100-A16 (Q96FQ6), appeared to increase when the cells were treated, with this type of proteins playing different types of roles on the epidermal differentiation complex, and thought to take part in cellular proliferation processes (Leśniak and Graczyk-Jarzynka 2015). Alternatively, proteins from the 14-3-3 family were also identified (14-3-3 protein zeta/delta, 14-3-3 protein sigma, 14-3-3 protein epsilon, 14-3-3 protein beta/alpha, 14-3-3 protein theta, 14-3-3 protein gamma), but only 14-3-3 protein sigma (P31947) showed differential results when compared with untreated cells, with a higher abundance of this protein observed in almost all the treatments applied (E1, E2 and MF1). This protein is directly related to keratinocyte differentiation and is involved in epidermal wound healing by stimulating the Akt/mTOR pathway, and could take part on the activation of p53/TP53 (Santoro et al. 2003; Yang et al. 2008). Another marker found was CD98hc (P08195), with this protein showing the highest variation when compared with control cells, being nearly twofold more abundant. CD98hc is essential for maintaining the integrity of the skin, cell proliferation, differentiation, fusion and adhesion (Devés and Boyd 2000). Furthermore, CD98 acts as an amino acid transporter to the inner cell, and so it could be enhancing the peptide and amino acid transport when the peptide mixtures are applied, enabling the access of peptides to the cell cytoplasm and thereby helping them to affect cellular gene expression and metabolism. In addition, CD98hc is strongly associated with skin homeostasis during ageing, and its deficiency produced a premature ageing skin phenotype due to the major ECM stiffness dysregulation (Tissot et al. 2018) and was described to be a cell proliferation booster, which could explain the increased proliferation observed in the treated keratinocytes (Cantor et al. 2009; Bajaj et al. 2016).

## Conclusions

Overall, this study reports on the wound healing promoting activity of different peptide mixtures from broccoli stems when they are applied to keratinocyte cell cultures. The BPs found in the treatments enhanced wound closure by overexpressing inflammatory response genes and cell growth promoting molecules related to cellular proliferation and wound repair. The E and MF extracts seemed to trigger similar fundamental pathways, as shown by the increased expression of TNF-α, and p53 but the reduction in AQP3 expression associated to cell protection from stress. However, the slight differences highlighted in Jun overexpression and pro-inflammatory responses were more evident in the MF extracts could indicate a different mechanism of regulation that was dependent on the protein source from which the peptides were extracted, which seemed to be related to Jun and AQP3 expression. More in-depth research is needed to fully understand the molecular mechanisms behind the cellular responses to these BP preparations and *in vitro* epidermis models.

## Supplementary Material

Supplemental Material

Supplemental Material
