# Supplementary material for: Bioactive peptides from broccoli stems strongly enhance regenerative keratinocytes by stimulating controlled proliferation
Source: Pharm Biol. 2022 Jan 27;60(1):235–46. doi: 10.1080/13880209.2021.2009522 (PMC8797740; doi:10.1080/13880209.2021.2009522)
Supplement: Supplemental Material [file IPHB_A_2009522_SM4551.docx]

**Supplementary Table 2.** Probable bioactive peptides with high similarities with bioactive peptides described in bibliography identified on the peptide mixtures (E and MF) and peptide function presented. The sequence underlined corresponds to similarities found with peptides in Plant Pep Data Base.

| **Peptide** **sequence** | **Presence** | | **ID Plant PepDB** | **Plant Source** | **Peptide Function** | **Peptide Function Description** |
| --- | --- | --- | --- | --- | --- | --- |
|  | **E** | **MF** |  |  |  |  |
| DAEGQDVLLFIDNIFR | x | x | [PPepDB_3170](http://223.31.159.8/PlantPepDB/pages/information.php?id=PPepDB_3170) | *Hordeum vulgare* | ACE-inhibitor | Shows angiotensin-I converting enzyme (ACE-I) inhibitory activity (Gangopadhyay et al. 2016; Moayedi et al. 2018). |
| DLPGLTDTEKPR | x |  | [PPepDB_3116](http://223.31.159.8/PlantPepDB/pages/information.php?id=PPepDB_3116) | *Solanum lycopersicum* | ACE-inhibitor/ Antioxidant |  |
| FEELNMDLFR | x |  | [PPepDB_3568](http://223.31.159.8/PlantPepDB/pages/information.php?id=PPepDB_3568) | *Oryza sativa* | Antimicrobial | Used in promotion of health and/or the treatment of diseases. It is also used as antimicrobial, endotoxin-neutralizing, arginine gingipain-inhibitory, and/or angiogenic activities (Taniguchi and Ochiai 2017). |
| MVNHFVQEFK | x |  | [PPepDB_3120](http://223.31.159.8/PlantPepDB/pages/information.php?id=PPepDB_3120) |  |  |  |
| LLHGVTIASGGV-  LPNINPVLLPK | x | x | [PPepDB_3221](http://223.31.159.8/PlantPepDB/pages/information.php?id=PPepDB_3221) | *Angelica sinensis* | Antioxidant | Used to delay aging process in *C. elegans* through antioxidant activities independent of dietary restriction (Wang et al. 2016). |
| MQIFVK | x | x | [PPepDB_3496](http://223.31.159.8/PlantPepDB/pages/information.php?id=PPepDB_3496) | *Solanum lycopersicum* | Antimicrobial | AMPs constitute a primitive mechanism of innate immunity and help as defense to protect their hosts from microbial attack (Hegedüs and Marx 2013). |
| LIFAGK | x |  | [PPepDB_3496](http://223.31.159.8/PlantPepDB/pages/information.php?id=PPepDB_3496) |  |  |  |
| TITLEVESSDTIDNVK | x | x | [PPepDB_3496](http://223.31.159.8/PlantPepDB/pages/information.php?id=PPepDB_3496) |  |  |  |
|  |  |  |  |  |  |  |

**Supplementary Table 3**. Keratinocyte protein profile of control cells and E1, E2, MF1 and MF2 treatments shorting by biological process protein Geneontology groups relative presence. Data are mean ± SE (n = 3). Statistical differences after Dunnet test analysis are indicated by asterisk indicating showing levels of significance (*:0.05; **: 0.005; ***: 0.0005; ****: 0.00005).

| **Biological process** | **C** | **E1** | **E2** | **MF1** | **MF2** |
| --- | --- | --- | --- | --- | --- |
| Cellular process | 17.96 ± 0.1 | 17.51 ± 0.1 | 17.64 ± 0.03 | 17.48 ± 0.03 | 16.29 ± 0.18*** |
| Biological regulation | 14.73 ± 0.06 | 14.51 ± 0.22 | 14.63 ± 0.17 | 14.45 ± 0.11 | 13.58 ± 0.4* |
| Metabolic process | 12.5 ± 0.18 | 12.03 ± 0.09 | 12.1 ± 0.25 | 12.13 ± 0.18 | 11.69 ± 0.16 |
| Localization | 10.36 ± 0.08 | 10.26 ± 0.38 | 10.47 ± 0.22 | 10.24 ± 0.34 | 10.16 ± 0.52 |
| Response to stimulus | 8.6 ± 0.05 | 9.15 ± 0.44 | 9.15 ± 0.18 | 9.43 ± 0.09 | 10.2 ± 0.09* |
| Cellular component | 8.46 ± 0.01 | 8.48 ± 0.06 | 8.34 ± 0.45 | 8.45 ± 0.07 | 8.2 ± 0.04 |
| Developmental process | 5.99 ± 0.16 | 6.31 ± 0.05 | 6.11 ± 0.19 | 5.8 ± 0.03 | 7.07 ± 0.46 |
| Interspecies interaction | 6.18 ± 0.13 | 5.7 ± 0.22 | 5.65 ± 0.27 | 5.68 ± 0.09 | 5.54 ± 0.11 |
| Immune system process | 4.13 ± 0 | 4.63 ± 0.27 | 4.61 ± 0.12 | 4.81 ± 0.14 | 4.92 ± 0.26 |
| Multicellular organismal | 3.37 ± 0.2 | 3.28 ± 0.05 | 3.2 ± 0.05 | 3.17 ± 0.01 | 3.82 ± 0.06 |
| Reproductive process | 1.9 ± 0.11 | 1.9 ± 0.16 | 1.86 ± 0.24 | 1.78 ± 0 | 2.01 ± 0.13 |
| Locomotion | 1.33 ± 0.2 | 1.52 ± 0.04 | 1.55 ± 0.02 | 1.65 ± 0 | 1.93 ± 0.19 |
| Biological adhesion | 0.81 ± 0.06 | 0.92 ± 0.04 | 0.89 ± 0.03 | 0.99 ± 0.03 | 1.19 ± 0.22 |
| Cell proliferation | 0.62 ± 0.04 | 0.51 ± 0.02 | 0.55 ± 0.02 | 0.66 ± 0.03 | 0.86 ± 0.08* |
| Rhythmic process | 0.52 ± 0.05 | 0.55 ± 0.06 | 0.55 ± 0.02 | 0.25 ± 0.25 | 0 ± 0 |
| Rulti-organsim process | 0.57 ± 0.01 | 0.6 ± 0.08 | 0.68 ± 0.11 | 0.72 ± 0.04 | 0.53 ± 0.06 |
| Cell killing | 0.43 ± 0.04 | 0.41 ± 0.02 | 0.5 ± 0.02 | 0.66 ± 0.03* | 0.64 ± 0.06* |
| Behavior | 0.47 ± 0.09 | 0.56 ± 0.12 | 0.5 ± 0.02 | 0.46 ± 0.05 | 0.1 ± 0.1 |
| Detoxification | 0.47 ± 0.09 | 0.42 ± 0.07 | 0.39 ± 0.01 | 0.47 ± 0.08 | 0.53 ± 0.06 |
| Signaling | 0.29 ± 0 | 0.41 ± 0.02** | 0.27 ± 0.01 | 0.26 ± 0.01 | 0 ± 0**** |
| Growth | 0.29 ± 0 | 0.33 ± 0.06 | 0.34 ± 0.05 | 0.4 ± 0.02 | 0.43 ± 0.04 |
| Luteolysis | 0 ± 0 | 0 ± 0 | 0 ± 0 | 0.07 ± 0.07 | 0.1 ± 0.1 |
| Regulation of heart rate | 0 ± 0 | 0 ± 0 | 0 ± 0 | 0 ± 0 | 0.21 ± 0.02**** |

**Supplementary Table 4.** Keratinocyte protein profile of control cells and E1, E2, MF1 and MF2 treatments shorting by molecular function Geneontology protein groups relative presence. Data are mean ± SE (n = 3). Statistical differences after Dunnet test analysis are indicated by asterisk indicating showing levels of significance (*:0.05; **: 0.005; ***: 0.0005; ****: 0.00005).

| **Molecular function** | **C** | **E1** | **E2** | **MF1** | **MF2** |
| --- | --- | --- | --- | --- | --- |
| Binding | 46.92 ± 0.2 | 46.64 ± 0.51 | 45.62 ± 1.25 | 45.36 ± 0.28 | 43.5 ± 0.73 |
| Catalytic activity | 21.81 ± 0.5 | 22.4 ± 1.08 | 22.56 ± 0.68 | 22.48 ± 0.51 | 22.65 ± 0.43 |
| Structural molecule activity | 10.39 ± 0.11 | 8.82 ± 1.37 | 8.48 ± 0.38 | 7.45 ± 0.13 | 7.41 ± 0.36 |
| Molecular function regulator | 7.56 ± 0.21 | 8.72 ± 0.67 | 8.83 ± 0.03 | 8.52 ± 0.19 | 9.53 ± 0.09***** |
| Transporter activity | 4.09 ± 0.42 | 3.83 ± 0.04 | 4.2 ± 0.03 | 4.54 ± 0.01 | 3.87 ± 0.02 |
| Transcription regulator activity | 2.44 ± 0.18 | 2.78 ± 0.3 | 3.02 ± 0.15 | 3.27 ± 0.14 | 2.95 ± 0.38 |
| Antioxidant activity | 2.18 ± 0.08 | 2.05 ± 0.16 | 2.14 ± 0.32 | 2.73 ± 0.3 | 3.59 ± 0.26***** |
| Protein folding chaperone | 1.93 ± 0.17 | 1.8 ± 0.14 | 2.27 ± 0.19 | 2.37 ± 0.28 | 2.35 ± 0.43 |
| Translation regulator activity | 2.18 ± 0.08 | 2.17 ± 0.04 | 2.27 ± 0.19 | 2.55 ± 0.11 | 3.27 ± 0.06****** |
| Superoxide dismutase copp | 0.26 ± 0.01 | 0.26 ± 0.02 | 0.31 ± 0.05 | 0.36 ± 0.02 | 0.28 ± 0.28 |
| Signalling receptor activity | 0.26 ± 0.01 | 0.38 ± 0.1 | 0.31 ± 0.05 | 0.36 ± 0.02 | 0.6 ± 0.04***** |
| Protein tag | 0 ± 0 | 0.14 ± 0.14 | 0 ± 0 | 0 ± 0 | 0 ± 0 |
