# Supplementary material for: Bioactive peptides from broccoli stems strongly enhance regenerative keratinocytes by stimulating controlled proliferation
Source: Pharm Biol. 2022 Jan 27;60(1):235–46. doi: 10.1080/13880209.2021.2009522 (PMC8797740; doi:10.1080/13880209.2021.2009522)
Supplement: Supplemental Material [file IPHB_A_2009522_SM4544.docx]

**Supplementary table 1**. Concentration of organic acids, sugars and others metabolites of protein hydrosilates extracts, total protein extract (E) and microsomal fraction (MF). Student’s *t*-test statistical analysis (*; P < 0.05 and **; P < 0.005). Data represents mean ± SE.

|  |  | **E** | **MF** |
| --- | --- | --- | --- |
|  |  | (mg metabolite/ g protein) | |
| **Organic acids** | 4-Aminohippurate | 11,14 ± 0,95* | 45,29 ± 0,16* |
|  | 4-Hydroxybenzoate | ND | 17,2 ± 0,23 |
|  | Formate | 12,03 ± 0,08** | 4,23 ± 0,03** |
|  | Fumarate | 7,73 ± 0,24* | 2,85 ± 0,04* |
| **Sugars** | Fructose | 589,51 ± 48,57 | 524,49 ± 8,56 |
|  | Glucose | 744,36 ± 0,29** | 511,96 ± 0,74** |
| **Others** | Choline | 39,6 ± 3,63* | 7,57 ± 0,11* |

The analysis has been performed by H-NMR following the protocol described by Young et al. (2004) and Choi et al. (2006). Samples were lyophilized and 50 mg of each sample were resuspended with 1:1 (MeOH: H_2_O). After sonication, samples were centrifuged. Liquid phase was taken and dried overnight using Speed-Vaccum at 30ºC. The samples were resuspended in 800 µL of KH_2_PO_4_ 100 mM at pH=6. Then, the samples were filtered with 0.45 µm nylon filters. Finally, 600 µL of each sample were passed to a NMR 5mm tube for its quantification through H-NMR.
